# Non-nosocomial healthcare-associated infective endocarditis in Taiwan: an underrecognized disease with poor outcome

**DOI:** 10.1186/1471-2334-11-221

**Published:** 2011-08-17

**Authors:** Kuan-Sheng Wu, Susan Shin-Jung Lee, Hung-Chin Tsai, Shue-Ren Wann, Jui-Kuang Chen, Cheng-Len Sy, Yung-Hsin Wang, Yu-Ting Tseng, Yao-Shen Chen

**Affiliations:** 1 Divison of Infectious Diseases, Department of Medicine, Kaohsiung Veterans General Hospital, 386 Ta-Chung 1st Road, Kaohsiung 813, Taiwan; 2 School of Medicine, National Yang-Ming University, No.155, Section 2, Linong Street, Taipei 112 Taiwan; 3 Graduate Institute of Environmental Education, National Kaohsiung Normal University, No.116, Heping 1st Road, Kaohsiung 802, Taiwan

## Abstract

**Background:**

Non-nosocomial healthcare-associated infective endocarditis (NNHCA-IE) is a new category of IE of increasing importance. This study described the clinical and microbiological characteristics and outcome of NNHCA-IE in Taiwan.

**Methods:**

A retrospective study was conducted of all patients with IE admitted to the Kaohsiung Veterans General Hospital in Kaohsiung, Taiwan over a five-year period from July 2004 to July 2009. The clinical and microbiological features of NNHCA-IE were compared to those of community-acquired and nosocomial IE. Predictors for in-hospital death were determined.

**Results:**

Two-hundred episodes of confirmed IE occurred during the study period. These included 148 (74%) community-acquired, 30 (15%) non-nosocomial healthcare-associated, and 22 (11%) nosocomial healthcare-associated IE. *Staphylococcus aureus *was the most frequent pathogen. Patients with NNHCA-IE compared to community-acquired IE, were older (median age, 67 vs. 44, years, *p *< 0.001), had more MRSA (43.3% vs. 9.5%, *p *< 0.001), more comorbidity conditions (median Charlson comorbidity index [interquartile range], 4[2-6] vs. 0[0-1], *p *< 0.001), a higher in-hospital mortality (50.0% vs. 17.6%, *p *< 0.001) and were less frequently recognized by clinicians on admission (16.7% vs. 47.7%, *p *= 0.002). The overall in-hospital mortality rate for all patients with IE was 25%. Shock was the strongest risk factor for in-hospital death (odds ratio 7.8, 95% confidence interval 2.4-25.2, *p *< 0.001).

**Conclusions:**

NNHCA-IE is underrecognized and carries a high mortality rate. Early recognition is crucial to provide optimal management and improve outcome.

## Background

Infective endocarditis (IE) is a serious disease with a high mortality rate, of up to 16-25% [1-6]. Major changes have occurred in the epidemiology of IE in association with advances in medical and surgical care. Rheumatic heart disease has been replaced as the major risk factor for IE by degenerative valve diseases, hemodialysis, prosthetic valve replacement, and intravenous drug use (IVDU) [2,7-10]. *Staphylococci*, instead of *Streptococci*, have become the most common pathogen in IE patients in recent series [7,8,11].

Nosocomial IE is associated with the highest mortality rate among all forms of IE [10,12]. Nosocomial acquisition is considered when IE occurs within the hospital or in recently hospitalized patients [12,13]. Within the past two decades more invasive procedures and life-support measures, previously limited to hospitals are now performed in chronic care facilities or at home. A new category of IE, non-nosocomial healthcare-associated IE (NNHCA-IE), was recently introduced to encompass these changes in medical practice. This new entity is defined as an episode of IE in out-patients who had extensive exposure to medical care [14-17].

NNHCA-IE has been reported to contribute to 9.3% of all IE episodes [15]. The highest percentage was noted in North America. A large prospective, multicenter, international study, the International Collaboration of Endocarditis-Prospective Cohort Study (ICE-PCS) [15], mainly focused on the Americas and Europe. Only a few study sites were located in Western, Southern and Southeastern Asia. A subgroup analysis for these regions has not as yet been reported [15]. Patients with NNHCA-IE were found to have clinical features and outcomes similar to those with nosocomial IE [14]. Because these infections occur outside the hospital, clinicians appear to be unaware of the need to identify these cases and provide optimal antimicrobial therapy.

To our knowledge, NNHCA-IE has not been reported previously in Eastern Asia (including China, Hong Kong, Taiwan, Japan, Mongolia, North Korea and South Korea). Among the studies of IE conducted in this region [4,5,18-23], only two included nosocomial IE. Nosocomial acquisition was found to be an independent factor for poor prognosis [4,18]. However, the definitions of nosocomial IE were not clearly defined in these studies. It is also unclear whether NNHCA-IE was adequately distinguished from community-acquired or nosocomial-acquired IE. To better characterize the prevalence, causative microorganisms, clinical features and outcome of non-nosocomial healthcare-associated IE in Eastern Asia, we conducted a five-year retrospective study of all cases of IE in a large medical center in southern Taiwan.

## Methods

### Patients

Medical records of patients more than 18 years old with IE who were admitted to the Kaohsiung Veterans General Hospital (KVGH) from July 2004 to July 2009, were reviewed retrospectively. KVGH is a 1,200-bed teaching hospital. It provides both primary and tertiary medical services in Kaohsiung, a city with 1.52 million residents located in southern Taiwan. Medical charts of patients who met the *International Classification of Diseases, Ninth Revision *(ICD-9) diagnostic codes for endocarditis (ICD-9 codes 421.0, 421.1, and 421.9) were reviewed by infectious disease specialists. Diagnosis of IE were confirmed based on the modified Duke criteria [24]. Only those with definite IE were included to the study. Patients with a relapse of IE, defined as isolation of the same pathogen within a six-month period [17], were excluded. A standardized case report form was designed to collect the clinical characteristics of the patients, including age, sex, clinical manifestations, predisposing heart conditions, underlying diseases, Charlson comorbidity index [25], risk behaviors, causative microorganisms and outcome. The study was approved by the KVGH Institutional Review Board and informed consent was waived because of the retrospective nature of the study, and lack of direct patient contact or intervention.

### Definitions

An IE episode was defined as non-nosocomial healthcare-associated when it occurred within 48 hours of admission in a patient fulfilling at least one of the following criteria: (1) had received intravenous therapy, wound care, specialized nursing care, hemodialysis or intravenous chemotherapy within 30 days prior to the onset of IE, (2) was hospitalized in an acute care hospital for 2 or more days within 90 days before the onset of IE, or (3) resided in a nursing home or long-term care facility before admission [14,16,17,26]. An IE episode was defined as nosocomial healthcare-associated if positive blood cultures were obtained from a patient hospitalized for more than 48 hours without relevant symptoms and signs on admission [14,16,17,26,27]. The infection was considered to be community-acquired when the symptoms and signs of IE occurred within 48 hours of admission in a patient not fulfilling the criteria for non-nosocomial healthcare-associated infection. IVDU-associated IE was defined as patients actively using illicit intravenous drugs at the time of infection. A predisposing heart condition was defined as a history of prosthetic cardiac valve replacement, previous bacterial endocarditis, congenital cardiac malformation, rheumatic and other acquired valvular dysfunction, hypertrophic cardiomyopathy or mitral valve prolapse with valvular regurgitation [28,29]. When a patient was suspected or diagnosed to have IE within the first 24 hours of medical access by clinicians, he was considered to be recognized on admission. Appropriate antibiotics were defined as having in vitro activities against the causative pathogens [30].

Presenting symptoms were divided into 5 symptomatic categories according to site: (1) constitutional including fever, fatigue and weight loss; (2) cardiopulmonary including dyspnea, orthopnea and chest pain; (3) neurological including change in consciousness, syncope and focal neurological deficits; (4) gastrointestinal including nausea, vomiting, abdominal pain and diarrhea; (5) musculoskeletal including focal arthralgia or myalgia, skin rash, subcutaneous abscess and cellulitis.

### Statistical analysis

In univariate analysis, categorical variables were compared using χ^2 ^test or Fisher's exact test as appropriate. Continuous variables were analyzed by Mann-Whitney U test. The result was considered to be significant if a two-tailed *p *value was less than 0.05. Variables with a *p *value of less than 0.05 in univariate analysis were included in multivariate logistic regression analysis by forward stepwise method using *p *values less than 0.05 as inclusion criterion and 0.1 as removal criterion. All data were analyzed by SPSS 17.0 for Windows (SPSS Inc. Chicago, USA).

## Results

During the five-year study period, 481 admissions with compatible ICD-9 codes for IE were reviewed. We identified 205 episodes of definite IE and 32 of possible IE. We excluded 5 patients with definite IE because they were considered to have had a relapse and all cases of possible IE. The final study population consisted of 192 patients with 200 episodes of definite IE, of which 168 (84%) were diagnosed by clinical and 32 (16%) by pathological criteria. There were 148 (74%) episodes of community-acquired and 52 (26%) healthcare-associated IE. Thirty (57.7%) of the 52 healthcare-associated IE were considered to be non-nosocomial. They comprised 15% of all IE episodes. There were 59 (29.5%) IVDU-associated IE episodes. All of these were community-acquired. The ten patients with IE and human immunodeficiency virus (HIV) infection were all IVDU-associated. Paravalvular abscess was noted in only one patient, whose IE was community-acquired. Overall, patients who were recognized as having NNHCA-IE more frequently received appropriate antibiotics within the first 24 hours of presentation than those who were not (77.6% vs. 37.6%, *p *< 0.001).

Of the 30 IE patients who met non-nosocomial healthcare-associated infection criteria, 14 were receiving regular hemodialysis prior to the onset of IE, 19 were recently hospitalized in acute care hospitals for 2 or more days (median duration from the time being discharged to the time IE symptoms began were 33 days, with an interquartile rage of 15-55 days), 4 were nursing home residents and 1 had received intravenous chemotherapy 7 days before the onset of IE.

### NNHCA-IE versus community-acquired IE

The thirty patients with NNHCA-IE were older (median age, 67 vs. 44, years, *p *< 0.001), more often female (43.3% vs. 18.9%, *p *= 0.006), and had more frequently co-morbidities (median Charlson comorbidity index [interquartile range], 4[2-6] vs. 0[0-1], *p *< 0.001) (Table 1). Up to 60% of NNHCA-IE patients had Charlson comorbidity indexes of 4 or more. NNHCA-IE was more often caused by *Staphylococci *(76.7% vs. 50.7%, *p *= 0.015), methicillin-resistant *Staphylococcus aureus *(MRSA) (43.3% vs. 9.5%, *p *< 0.001), and less commonly by *Streptococci *(13.3% vs. 34.5%, *p *= 0.029). Patients with NNHCA-IE had a significantly higher in-hospital mortality rate (50.0% vs. 17.6%, *p *< 0.001).

**Table 1:** Comparison of clinical characteristics and outcomes of NNHCA-IE with community-acquired IE and nosocomial IE among patients admitted to the Veterans General Hospital Kaohsiung, Taiwan over a five-year period (2004 to 2009)

	All	Group A: NNHCA-IE n = 30	Group B: Community-acquired IE n = 148	Group C: Nosocomial IE n = 22	Group A vs B *p *value	Group A vs C *p *value
Age, years						
Median (range)	50 (19-92)	67 (45-84)	44 (19-88)	78 (43-92)	< 0.001	0.011
Women, n (%)	48 (24.0)	13 (43.3)	28 (18.9)	7 (31.8)	0.004	0.399
Prosthetic valve, n (%)	18 (9.0)	2 (6.7)	15 (10.1)	1 (4.5)	0.741	1.000
Underlying diseases, n (%)						
Predisposing heart condition	49 (24.5)	6 (20.0)	40 (27.0)	3 (13.6)	0.423	0.717
Diabetes mellitus	52 (26.0)	17 (56.7)	25 (16.9)	10 (45.5)	< 0.001	0.424
Hypertension	54 (27.0)	20 (66.7)	26 (17.6)	8 (36.4)	< 0.001	0.030
Heart failure	35 (17.5)	4 (13.3)	25 (16.9)	6 (27.2)	0.789	0.290
HIV infected	10 (5.0)	0 (0)	10 (6.8)	0 (0)	0.216	NA
IVDU	59 (29.5)	0 (0)	59 (40.0)	0 (0)	< 0.001	NA
Charlson comorbidity index, median (IQR)	1 (0-2)	4 (2-6)	0 (0-1)	2 (1-4)	< 0.001	0.06
0-1	132 (66.0)	3 (10.0)	121 (81.8)	8 (36.4)		
2-3	34 (17.0)	9 (30.0)	19 (12.8)	6 (27.3)		
≧4	34 (17.0)	18 (60.0)	8 (5.4)	8 (36.4)		
Dental procedure	9 (4.5)	0 (0)	8 (5.4)	1 (4.5)	0.193	0.423
Symptom duration, days, median (IQR) ^1^	5 (3-15)	4 (2-7)	7 (3-20)	NA	0.031	NA
Symptoms, n (%) ^1^						
Constitutional	142 (71.0)	26 (86.7)	104 (70.3)	12 (54.5)	0.065	0.010
Cardiopulmonary	65 (32.5)	6 (20.0)	50 (33.8)	9 (40.9)	0.138	0.100
Neurological	47 (23.5)	4 (13.3)	40 (27.0)	3 (13.6)	0.113	1.000
Gastrointestinal	14 (7.0)	3 (10.0)	8 (5.4)	3 (13.6)	0.399	0.689
Musculoskeletal	24 (12.0)	5 (16.7)	19 (12.8)	0 (0)	0.563	0.065
Signs, n (%) ^2^						
Fever	165 (82.5)	28 (93.3)	121 (81.8)	16 (72.7)	0.174	0.058
Heart murmur	83 (41.5)	10 (33.3)	68 (45.9)	5 (22.7)	0.204	0.404
Skin lesions	23 (11.5)	3 (10.0)	20 (13.5)	0 (0)	0.770	0.253
Embolism	98 (49.0)	11 (36.7)	82 (55.4)	5 (22.7)	0.061	0.282
Altered consciousness	34 (17.0)	4 (13.3)	28 (18.9)	2 (9.1)	0.468	1.000
Shock	20 (10.0)	2 (6.7)	16 (10.8)	2 (9.1)	0.742	1.000
Recognition on admission, n (%) ^3^	68/162 (42.0)	5/30 (16.7)	63/132 (47.7)	NA	0.002	NA
Appropriate antibiotics on admission, n (%) ^4^	94/165 (57.0)	13/30 (43.3)	81/135 (60.0)	NA	0.095	NA
Outcome						
Antibiotic treatment duration, median days (IQR, n) ^5^	32 (28-41, n = 138)	28 (27-40, n = 17)	32 (28-42, n = 113)	34 (30-40, n = 8)	0.258	0.277
Hospital stay, median days (IQR, n) ^5^	37 (29-48, n = 131)	46 (30-65, n = 17)	37 (29-45, n = 114)	NA	0.181	NA
In-hospital death	50 (25.0)	15 (50.0)	26 (17.6)	9 (40.9)	< 0.001	0.516

^1 ^Duration from the time of onset of symptoms to admission to hospital.

^2 ^Includes all symptoms and signs of IE from their onset to diagnosis.

^3 ^The number of patients who were suspected or diagnosed to have IE within the first 24 hours of medical access. Exclude patients who were referred from other hospitals with a definite diagnosis already.

^4 ^The number of patients who received antibiotics with in vitro activities against the pathogens within the first 24 hours of medical access.

^5 ^Only patients who have received a complete course of antibiotics for IE were included.

NNHCA-IE, non-nosocomial healthcare-associated infective endocarditis; HIV, human immunodeficiency virus; NA, not applicable; IVDU, intravenous drug user; IQR, interquartile range

### NNHCA-IE versus nosocomial IE

The twenty two patients with nosocomial IE were even older (median age, 78 vs. 67, years, *p *< 0.011). There was no significant difference in gender (female, 43.3% vs. 31.8%, *p *= 0.40), Charlson comorbidity index (median [interquartile range], 4[2-6] vs. 2[1-4], *p *= 0.06), *Staphylococcal *infection (76.7% vs. 77.3%, *p *= 1.00), *Streptococcal *infection (13.3% vs. 9.1%, *p *= 1.00) and in-hospital mortality rate (50.0% vs. 40.9%, *p *= 0.52) between patients with NNHCA-IE and nosocomial IE.

### Microbiological findings

The microbiological findings according to the site of origin are shown in Table 2. *Staphylococcus aureus *was the most common causative microorganism except for non-IVDU community-acquired IE. *Streptococci *remained the most predominant causative pathogen in this group. There were 8 culture-negative IE patients, and 3 of them had been exposed to antimicrobial therapy before the blood cultures were drawn for culture.

**Table 2:** Microorganisms causing 200 episodes of IE among patients admitted to the Veterans General Hospital Kaohsiung, Taiwan over a five-year period (2004 to 2009)

Pathogens	All	Community-acquired IE		Healthcare-associated IE	
	N = 200	non-IVDU n = 89	IVDU n = 59	non-nosocomial n = 30	nosocomial IE n = 22
*Staphylococci*, n (%)	115 (57.5)	21 (23.6)	54 (91.5)	23 (76.7)	17 (77.3)
MSSA	65	15	42	8	0
MRSA	44	2	12	13	17
Coagulase-negative *Staphylococci*	6	4	0	2	0
*Streptococci*, n (%)	57 (28.5)	50 (56.2)	1 (1.7)	4 (13.3)	2 (9.1)
Viridans streptococci	38	35	0	3	0
*Streptococcus bovis*	9	8	0	1	0
*Streptococcus agalactiae*	5	3	1	0	1
*Streptococcus pyogenes*	1	1	0	0	0
*Streptococcus pneumoniae*	1	1	0	0	0
NVS	3	2	0	0	1
*Enterococci*, n (%)	9 (4.5)	5 (5.6)	0 (0)	3 (10.0)	1 (4.5)
*Enterococcus faecalis*	8	5	0	2	1
*Enterococcus faecium*	1	0	0	1	0
Gram negative bacilli, n (%)	3 (1.5)	0 (0)	2 (3.4)	0 (0)	1 (4.5)
*Pseudomonas aeruginosa*	1	0	1	0	0
*Chryseobacterium indologenes*	1	0	0	0	1
*Enterobacter cloacae*	1	0	1	0	0
HACEK group, n (%)	2 (1.0)	2 (2.2)	0 (0)	0 (0)	0 (0)
*Haemophilus actinomycetemcomitans*	1	1	0	0	0
*Haemophilus parainfluenzae*	1	1	0	0	0
Others, n (%)	6 (3.0)	5 (5.6)	1 (1.7)	0 (0)	0 (0)
*Rothia dentocariosa*	2	1	1	0	0
*Finegoldia magna*	1	1	0	0	0
*Lactococcus lactis*	1	1	0	0	0
*Lactococcus gravieae*	1	1	0	0	0
*Candida albicans*	1	1	0	0	0
Culture negative, n (%)	8 (4.0)	6 (6.7)	1 (1.7)	0 (0)	1 (4.5)

IE, infective endocarditis; IVDU, intravenous drug user; MSSA, methicillin-susceptible *Staphylococcus aureus*; MRSA, methicillin-resistant *Staphylococcus aureus*; NVS, nutritionally variant streptococci; HACEK, *Haemophilus *species, *Actinobacillus actinomycetemcomitans*, *Cardiobacterium hominis*, *Eikenella corrodens*, *Kingella *species

### Recognition of NNHCA-IE

NNHCA-IE was less often clinically recognized on admission than community-acquired IE (5/30, 16.7% vs. 63/132, 47.7%, *p *= 0.002). NNHCA-IE were initially misdiagnosed as skin and soft tissue infection (8 episodes), catheter-related infection (5), pneumonia (4), urinary tract infection (4), fever of undetermined focus (3) and meningitis (1). Only 43.3% of these patients received appropriate antibiotics within the first 24 hours of admission. Delay of appropriate antimicrobial therapy was significantly associated with in-hospital death in community-acquired IE patients (27.8% vs. 9.9%, *p *= 0.007), but not in NNHCA-IE patients (52.9% vs. 46.2%, *p *= 0.713).

Three significantly different clinical characteristics were identified for NNHCA-IE among all IE episodes acquired outside the hospital using multivariate logistic regression analysis, as shown in Table 3. Having a Charlson comorbidity index of 2 or more was the most significant predictor (odds ratio [OR] 27.5, 95% confidence interval [CI] 6.9-110.3, *p *< 0.001), followed by MRSA infection and having a past history of hypertension.

**Table 3:** Significant differences in the clinical characteristics of patients with NNHCA-IE compared to community-acquired IE, as determined by multivariate analysis

	NNHCA-IE	Community-	Univariate		Multivariate ^1^	
			
	n = 30	acquired IE n = 148	OR (95%CI)	*p *value	OR (95%CI)	*p *value
Age ≧ 65 years, n (%)	19 (63.3)	27 (18.2)	7.7 (3.3-18.1)	< 0.001		
Women, n (%)	13 (43.3)	28 (18.9)	3.3 (1.4-7.5)	0.004		
Charlson comorbidity index ≧ 2 n (%)	27 (90.0)	27 (18.2)	40.3 (11.4-142.7)	< 0.001	27.5 (6.9-110.3)	< 0.001
Diabetes mellitus, n (%)	17 (56.7)	25 (16.9)	6.4 (2.8-14.9)	< 0.001		
Hypertension, n (%)	20 (66.7)	26 (17.6)	9.4 (3.9-22.4)	< 0.001	7.5 (2.2-25.1)	0.001
Symptoms duration ≦ 7 days n (%)	25 (83.3)	95 (64.2)	2.8 (1.0-7.7)	0.041		
*Staphylococci *infection, n (%)	23 (76.7)	75 (50.7)	3.2 (1.3-7.9)	0.009		
MRSA infection, n (%)	13 (43.3)	14 (9.5)	7.3 (3.0-18.1)	< 0.001	12.2 (2.9-51.5)	0.001
*Streptococci *infection, n (%)	4 (13.3)	51 (34.5)	0.3 (0.1-0.9)	0.022		

^1 ^Adjusted for age and sex

NNHCA-IE, non-nosocomial healthcare-associated infective endocarditis; OR, odds ratio; CI, confidence interval; MRSA, methicillin-resistant *Staphylococcus aureus*

### Risk factors for in-hospital mortality

Twenty five percent of all the patients (n = 50) died during hospitalization. Patients with NNHCA-IE had the highest in-hospital mortality rate (15/30, 50.0%), followed by nosocomial healthcare-associated IE (9/22, 40.9%) and community-acquired IE (26/148, 17.6%). In-hospital mortality rate was significantly higher in NNHCA-IE compared to community-acquired IE (*p *< 0.001). Risk factors significantly associated with in-hospital death according to univariate and multivariate analysis are shown in Table 4. In multivariate logistic regression analysis, the significant risk factors for in-hospital death, after adjusting for age and sex, included shock (odds ratio 7.8, 95% confidence interval 2.4-25.2, *p *< 0.001), non-nosocomial healthcare-associated infection, alteration in consciousness, delay of appropriate antibiotics use and *Staphylococci *infection.

**Table 4:** Predictors for in-hospital death in 200 episodes of IE among patients admitted to the Veterans General Hospital in Kaohsiung, Taiwan over a five-year period (2004 to 2009) as determined by multivariate analysis

Risk factors	All	Death	Univariate		Multivariate ^1^	
			
			OR (95%CI)	*p *value	OR (95%CI)	*p *value
Age ≧ 65 years	64	26	3.2 (1.6-6.2)	< 0.001		
Female gender	48	20	2.9 (1.4-5.8)	0.002		
NNHCA IE	30	15	3.9 (1.7-8.6)	0.001	6.0 (2.3-16.1)	< 0.001
Nosocomial IE	22	9	2.3 (0.9-5.8)	0.068		
Prosthetic valve	18	9	3.4 (1.3-9.2)	0.019		
*Staphylococci*	115	35	2.0 (1.0-4.0)	0.039	3.0 (1.1-8.0)	0.027
*Streptococci*	57	6	0.3 (0.1-0.7)	0.003		
Underlying conditions						
Diabetes mellitus	52	24	4.0 (2.0-8.0)	< 0.001		
Hypertension	54	18	1.8 (0.9-3.5)	0.098		
Predisposing heart condition	49	14	1.3 (0.6-2.6)	0.506		
IVDU	59	10	0.5 (0.2-1.1)	0.089		
Charlson comorbidity index ≧ 2	68	30	4.4 (2.3-8.7)	< 0.001		
Symptoms						
Constitutional	142	34	0.8 (0.4-1.7)	0.589		
Cardiopulmonary	65	18	1.2 (0.6-2.4)	0.542		
Neurological	47	15	1.6 (0.8-3.2)	0.211		
Gastrointestinal	14	4	1.2 (0.4-4.1)	0.749		
Musculoskeletal	24	5	0.8 (0.3-2.2)	0.615		
Signs						
Fever	165	41	1.0 (0.4-2.2)	0.914		
Heart murmur	83	19	0.8 (0.4-1.6)	0.562		
Alteration in consciousness	34	15	3.0 (1.4-6.4)	0.005	3.9 (1.4-11.1)	0.012
Shock	20	13	7.2 (2.7-19.3)	< 0.001	7.8 (2.4-25.2)	0.001
Unrecognition on admission ^2^	94/162	25	1.3 (0.6-2.7)	0.509		
Delay of appropriate antibiotics ^3^	71/165	24	2.9 (1.4-6.2)	0.004	3.7 (1.5-9.1)	0.004

^1 ^Adjusted for age and sex

^2 ^Patients who were not suspected or diagnosed to have IE within the first 24 hours of medical access.

^3 ^Patients didn't receive antibiotics with in vitro activities against the pathogens within the first 24 hours of medical access.

IE, infective endocarditis; OR, odds ratio; CI, confidence interval; NNHCA, non-nosocomial healthcare-associated; IVDU, intravenous drug user

## Discussion

Non-nosocomial healthcare-associated infective endocarditis (NNHCA-IE) is a new category of IE that often goes unrecognized in Taiwan. This is the first study to describe the clinical characteristics and outcomes of NNHCA-IE in Eastern Asia and emphasize the differences between NNHCA-IE and community-acquired IE. This study also describes the changing distribution of the causative microorganisms of IE in Taiwan. Five independent predictors for in-hospital death were identified.

In our study clinical features and outcomes of patients with NNHCA-IE were very different from those with community-acquired IE, but similar to those with nosocomial IE. The findings were comparable to those in a previous study[14]. However, it is easy to mistake NNHCA-IE for community-acquired IE, since both appear to be acquired outside the hospital. Symptoms and signs of IE patients at presentation were not helpful in distinguishing between these two categories of IE. We identified 3 independent predictors for all IE episodes acquired out of the hospital to be non-nosocomial healthcare-associated, including having a Charlson comorbidity index of 2 or more, having a history of hypertension and MRSA infection. Clinicians should be alert to the possibility of NNHCA-IE when patients with IE present with these 3 different clinical features.

Early recognition of IE enables clinicians to initiate appropriate antimicrobial therapy early. However, only 15.4% of NNHCA-IE patients were suspected or diagnosed to have IE on admission. The percentage was significantly lower than that of community-acquired IE patients. There are two possible explanations. First, patients with NNHCA-IE were less likely to present with embolic phenomena or a heart murmur than those with community-acquired IE. Second, many clinicians are not familiar with the atypical presentations of the new category of IE.

Only 43.3% of patients with NNHCA-IE received appropriate antimicrobial therapy on the first day. Delay of appropriate antimicrobial therapy was an independent predictor for in-hospital death in all IE patients, but not in NNHCA-IE patients. We propose two possible explanations. First, patients with NNHCA-IE had more underlying diseases, which were mainly responsible for the poor outcome and masked the impact of inappropriate antimicrobial therapy. Second, the case number of NNHCA-IE in our series was small. Further larger, prospective studies may be needed to evaluate the impact of inappropriate antimicrobial therapy on NNHCA-IE.

NNHCA-IE is an emerging category of IE. In a population-based study of IE, 50% of IE patients between 2001 and 2006 were non-nosocomial healthcare-associated. The percentage was higher than that of community-acquired IE (42.5%) and nosocomial IE (7.5%) [31]. In our study, NNHCA-IE accounted for 15% of all IE episodes. This is similar to the rates in the ICE-PCS study (254/1622, 15.7%) [14]. The rates of NNHCA-IE in Taiwan were lower than those in North America, but higher than South America and Europe [15]. Non-nosocomial acquisition accounted for 58% of health care-associated IE episodes at our medical center. This is slightly higher than the 46% reported in ICE-PCS [14]. Taiwan is a country in which 99.5% of the population is covered by national health insurance. This has augmented access to medical clinics, regional hospitals, hemodialysis and chronic care centers. Improved access to medical care, accompanied by advances in invasive medical procedures, increased the proportion of outpatient procedures and care, which further contributed to the frequency of NNHCA-IE.

There are only limited reports of the microbiological epidemiology of IE in Eastern Asian countries during the past decade [4,5,19-21,23]. Some report *Staphylococci *[19-21], others found *Streptococci *[4,5,23] to be most common pathogens. None of these studies included the underlying risk factors for the new classification of IE. We found *Staphylococcus aureus *to be the most frequent causative pathogen, except for non-IVDU community-acquired IE, where viridans *Streptococci *remain the predominant pathogen. Our findings are in accordance with those in the ICE-PCS [14]. The major difference was that our study population had a relatively low proportion of *Enterococci *(4.5%) compared to 8.0%-19.5% in western countries [1,2,7,8,11,15,32,33].

We identified 5 risk factors for in-hospital death by multivariate logistic regression analysis. Most are known independent predictors of mortality. These included shock [2,5], non-nosocomial healthcare-associated infection, alteration in consciousness [4,5], delay of appropriate antibiotics use [34,35] and *Staphylococci *infection [5,14]. Healthcare-associated infection has been identified to be associated with poor outcome in IE [14]. In our study, patients with non-nosocomial acquisition had an even higher risk for in-hospital death. Patients with NNHCA-IE also had many comorbid conditions requiring frequent medical care and exposure. Increased awareness of clinicians to this disease entity could reduce mortality associated with NNHCA-IE.

This current study has several limitations. First, the retrospective design of the current study may have missed some clinical features such as new heart murmurs or ascertainment of healthcare-associated criteria in some of the community-acquired IE cases. Second, we were unable to determine long-term survival and emergence of further complications following discharge from the hospital. Third, it was conducted in a single teaching hospital. Nevertheless, the age and sex of patients were comparable to those in previous studies [4,5,19], including the large Taiwan nationwide population-based IE study that included 7240 patients [5].

## Conclusions

In conclusion, NNHCA-IE accounted for 15% of IE in Taiwan, but was frequently unrecognized. It was associated with a higher in-hospital mortality rate than community-acquired IE. Increased awareness of this new category of IE is crucial to optimize therapy and improve the outcome associated with this entity.

## Competing interests

The authors declare that they have no competing interests.

## Authors' contributions

KSW participated in the design of the study, performed data analysis and drafted the manuscript. SSJL participated in the design of the study, assisted in data interpretation and gave crucial revision to this manuscript. HCT participated in the design of the study and gave crucial revision to this manuscript. SRW gave critical suggestions to the designs of the study and the manuscript. JKC, CLS, YHW and YTT participated in data collection, interpretation and analysis. YSC supervised the research group and gave final approval of the version to be submitted. All authors read and approved the final manuscript.

## Pre-publication history

The pre-publication history for this paper can be accessed here:

http://www.biomedcentral.com/1471-2334/11/221/prepub
